# Case Report: Successful non-operative management of spontaneous splenic rupture in a patient with babesiosis

**DOI:** 10.1186/1749-7922-6-4

**Published:** 2011-01-20

**Authors:** William D Tobler, Deborah Cotton, Timothy Lepore, Suresh Agarwal, Eric J Mahoney

**Affiliations:** 1 Boston University Medical Center 850 Harrison Avenue Dowling 2 South Boston Ma, 02118 USA; 2 Nantucket Cottage Hospital 57 Prospect St Unit 1 Nantucket Ma, 02554 USA

## Abstract

**Background:**

Babesiosis is a zoonotic disease transmitted by the *Ixodes *tick species. Infection often results in sub-clinical manifestations; however, patients with this disease can become critically ill. Splenic rupture has been a previously reported complication of babesiosis, but treatment has always led to splenectomy. Asplenia places a patient at greater risk for overwhelming post-splenectomy infection from encapsulated bacteria, Lyme disease, Ehrlichia as well as *Babesia microti*. Therefore, avoiding splenectomy in these patients must be considered by the physician; particularly, if the patient is at risk for re-infection by living in an endemic area.

**Case Presentation:**

A 54 year-old male from the northeast United States presented with left upper quadrant abdominal pain associated with fever, chills, night sweats and nausea. A full evaluation revealed active infection with *Babesia microti *and multiple splenic lacerations. This patient was successfully treated with appropriate pharmacological therapy and non-operative observation for the splenic injury.

**Conclusion:**

Patients diagnosed with *Babesia microti *infection are becoming more common, especially in endemic areas. Although clinical manifestations are usually minimal, this infection can present with significant injuries leading to critical illness. We present the successful non-operative treatment of a patient with splenic rupture due to babesiosis infection.

## Introduction

Babesiosis, most commonly caused by *Babesia microti *infection is becoming a more prevalent disease. In the United States, Martha's Vineyard, Nantucket, Shelter Island, and Long Island are considered some of the endemic areas for this infection[1]. Disease manisfestations range from subclinical to severe critical illness. Spontaneous splenic rupture is a rare complication that has been previously documented leading to emergent splenectomy in all cases[2,3]. However, asplenia places the patient at an increased risk for overwhelming post-splenectomy infection and critical illness. Therefore, splenic preservation should be a priority when treating a patient with splenic rupture following babesiosis infection, particularly for those residing in endemic areas. Prior to this case presentation, successful non-operative treatment following splenic rupture due to babesiosis has not been reported.

## Case Report

A 54 year-old male presented to a small community hospital in eastern Massachusetts with complaints of dull left upper quadrant abdominal pain, fever of 102.3 degrees Fahrenheit, nausea, chills, night sweats and dark urine for 48 hours. The patient recently traveled in Maine, northeastern Massachusetts, and Nantucket Island, Massachusetts. During his travels these symptoms progressed prompting him to seek medical attention. The patient was noted to be leukopenic, thrombocytopenic, and anemic with peripheral blood smear showing ring forms consistent with *Babesia microti*. A computed tomography (CT) scan was performed revealing perisplenic fluid in the subphrenic region with an upper limits of normal-sized spleen, and a small amount of free fluid in the pelvis suggesting hemoperitoneum. The patient was started on atovaquone and azithromycin and transferred to the Boston Medical Center. Upon presentation the patient reported improved abdominal pain.

The patient's past medical history is significant for Lyme disease, left rotator cuff surgery 8 weeks prior to presentation, and a laparoscopic right inguinal hernia repair. He denied any medications. The patient reported travel in the upper east coast of the United States but denied recent travel beyond that. Of note, he has two homes both of which are in endemic areas of tick-borne illnesses. The patient denied smoking, significant alcohol use, and drug use.

On physical exam, vitals signs were as follows: temperature 99.3 degrees (F), pulse 94 beats per minute, blood pressure 133/80 mmHg, respiratory rate 20 breaths per minute, oxygen saturation 99% on room air. In general, the patient appeared pale but was awake, alert, and oriented to person, place, and time. On inspection, the abdominal exam revealed no rashes and negative Cullen and Grey-Turner signs. There was minimal tenderness to palpation of the left lower quadrant; otherwise, the abdominal exam was benign. Furthermore, the remainder of the physical exam was unremarkable.

Laboratory values were significant for white blood cell count 4.0 × 10^9^/L, hemoglobin 102 g/L (10.2 g/dL), hematocrit 28.8%, platelet count 26.0 × 10^9^/L, bilirubin total 32.49 μmol/L (1.9 mg/dL), bilirubin direct 17.1 μmol/L (1.0 mg/dL), LDH 591 units/L, ALT 180 units/L, AST 68 units/L, and alkaline phosphatase 116 units/L.

**Figure 1:**
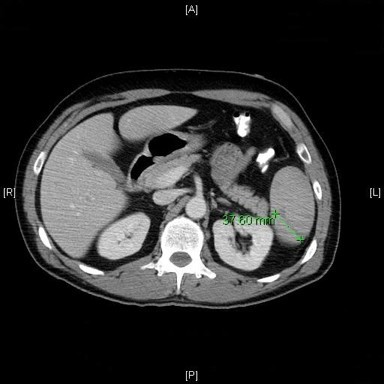
**Abdominal CT scan**. The CT scan from this patient shows a mildly enlarged spleen measuring 14 cm in longitudinal dimension. He had multiple splenic lacerations however, and this slice shows a 3.7 cm transverse splenic laceration.

A repeat CT scan performed showed the spleen measured 14 cm in longitudinal length with multiple lacerations (the largest extending near the hilum), and perisplenic/perihepatic/peripelvic hemorrhage (Figure 1). Infectious Disease (ID) and General Surgery were consulted for further evaluation. ID reviewed the peripheral blood smear and confirmed the presence of Babesiosis with 3% parasitemia. Atovaquone and azithromycin were continued with the addition of doxycycline for presumptive coverage of Lyme disease and Ehrlichiosis. The patient was admitted to the surgical intensive care unit for expectant management of the splenic injury which included bed rest, serial abdominal exams, serial hemoglobin/hematocrit checks, and platelet transfusion to a goal of greater than 50.0 × 10^9^/L.

Non-operative course of management was chosen for several reasons. First, the patient was minimally symptomatic by the time of transfer with hemodynamically normal vital signs. Second, the parasite count was 3% indicating a high likelihood of prompt, successful response to pharmacological therapy. Lastly, the patient has a history of Lyme disease, and he resides in a highly endemic region for tick-borne diseases. It was the belief of the team that the patient would therefore be at significant risk for additional tick-borne illnesses in the future, and if infected again would have a higher risk of mortality if he were asplenic.

Blood cultures and DNA polymerase chain reaction (PCR) studies were sent for Babesiosis, Lyme disease, and Ehrlichiosis. Babesiosis serum IgG was low/normal and IgM was positive, which was interpreted as equivocal; however, *Babesia *PCR was positive for active infection. *Borellia *species PCR was negative and *Ehrlichia chaffensis *IgG/IgM antibodies and PCR were also negative.

The patient was observed in the hospital for four days with improved symptoms each day. At the time of discharge his leukopenia had resolved, hemoglobin increased to 103 g/L (10.3 g/dL) from a low of 85 g/L (8.5 g/dL). Platelets increased to 439.0 × 10^9^/L from a low of 26.0 × 10^9^/L status post transfusion of 15 units, and his bilirubin (direct and indirect) levels were also normal at discharge. The patient received a 10-day course of antibiotics in total. At his follow up appointment the patient was doing well and deemed appropriate to resume normal activity.

## Discussion

*Babesia *infection was first described in cattle by Babes in 1888, and the first human case described by Skrabalo in 1957[4,5]. *Babesia *is most commonly caused by *Babesia microti *infection transmitted by *Ixodes scapularis*, which is endemic in the northeast United States[6]. Reports of babesiosis have also come from Minnesota, Wisconsin, and outside of the United States in Europe and Asia[2,7-9]. The European infection however is most often caused by *Babesia divergens*[10]. In the United States, the geographical distribution of babesiosis is similar to Lyme disease, which is transmitted by the same tick, *Ixodes scapularis*. Although less widespread than Lyme disease, the dissemination of babesiosis is currently increasing. This may be due to the growth of the white-tailed deer and white-footed mouse population or simply due to increased awareness and reporting of the disease[6,11]. In addition to tick transmission, babesiosis can spread transplacentaly and through blood transfusions[12,13].

Clinical presentation ranges from the asymptomatic patient to the more critically ill patient. The intermediate disease includes nonspecific viral-like symptoms such as chills, sweats, headache, arthralgia, anorexia, cough, and nausea. On physical exam patients can present with splenomegaly or hepatomegaly. Symptoms in more severe disease include jaundice, retinal infarct, ecchymoses, congestive heart failure, disseminated intravascular coagulation, liver and renal failure, and splenic rupture[6,14]. Common laboratory findings consist of thrombocytopenia, normal to decreased leukocyte count, and hemolytic anemia[14]. The most severe infections occur in the elderly, immunocomprimised, or splenectomized patients[10].

**Figure 2:**
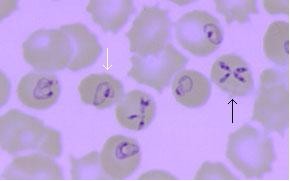
**Peripheral blood smear**. White arrow indicates pleomorphic, ring like structures often found with *Babesia *infection resembling early forms of malarial parasites such as Plasmodium falciparum. Black arrow shows the classic arrangement of 4 rings called the Maltese cross which is pathognomonic for *Babesia *infection. Image provided courtesy of Daniele Focosi MD, University of Pisa, Italy.

Diagnosis is determined by several methods. Microscopic identification is performed using Wright's or Giemsa stain which identify the *Babesia microti *organism[10]. A common morphology observed on these stains is a ring-form which has low specificity resembling the classic "signet rings" seen in malaria (white arrow, Figure 2). A pathognomonic but rare microscopic form is the Maltese cross (black arrow, Figure 2)[14,15]. Confirmatory tests include serology and PCR. Serology is utilized to identify positive IgG and IgM titers. PCR is more specific and sensitive, and is suggested when blood smears are non-conclusive[6].

The treatment of babesiosis traditionally consisted of clindamycin and quinine, but this therapy has multiple side effects including tinnitus, vertigo, and gastrointestinal upset[14]. Data from 2000 shows that mild to moderate disease can be treated with atovaquone and azithromycin for 7 to 10 days with comparable results and less side effects[16]. If there is no response to this therapy or the disease is severe then the recommendation is to transition medical therapy back to clindamycin and quinine[6,17]. Furthermore, exchange red blood cell transfusion is an option in patients with severe parasitemia (>5-10%) or if there is pulmonary, renal, or hepatic compromise[6,14,18,19].

Splenic injury is an uncommon complication of *Babesia *infection. There are several reports of splenic rupture as well as splenic infarction in the literature[2,3,20]. However, there are no reports describing successful non-operative management of significant rupture. Our institution has treated several patients in the past with splenic lacerations. Of these cases, one was successfully treated with splenic artery embolization and others with splenectomy. Two case reports previously published present a 61 year-old male and a 56 year-old male infected with babesiosis that were initially treated with observation and antibiotic therapy alone. However, both patients developed acute abdominal pain requiring further work-up. CT scans demonstrated splenic laceration in both patients, and they subsequently underwent emergent splenectomy due to worsening hemodynamic instability. Parasite count was noted to be 5% for the 61 year-old male, and not reported for the other[2,3]. In comparison to the two patients requiring operative invention, our patient had a slightly lower parasite count and received platelet transfusions. He was diagnosed early in his hospital course with a splenic rupture and was aggressively monitored in the surgical intensive care unit with serial abdominal exams.

The mechanism of splenic rupture is not entirely clear but may be a result of phagocytosis of *Babesia*-infected erythrocytes by splenic histiocytes in addition to sequestration of platelets causing thrombocytopenia. This process leads to rapid splenomegaly and eventual splenic rupture[2]. Splenomegaly was reported in only one of the previously published case reports; therefore, a benign abdominal exam cannot exclude splenic injury. Thus awareness and recognition of this complication may allow for early clinical management that may prevent splenectomy in select cases. This is important, particularly in patients living in endemic areas, because asplenia places a patient at greater risk for overwhelming post-splenectomy infection from encapsulated bacteria, Lyme disease, Ehrlichia as well as *Babesia*[10].

In asplenic patients, routine screening for *Babesia *may be indicated for those living in endemic areas[1]. Patients with babesiosis should also be screened for Lyme disease and Erlichiosis at the time of infection because co-infection often manifests as more severe disease[10].

## Conclusion

The incidence of babesiosis infection is increasing throughout the United States. This disease often presents with mild to moderate symptoms, but can rapidly progress to significant injury including splenic rupture. Early diagnosis, close observation, and platelet transfusions allow for effective and successful non-operative treatment for splenic rupture. Most importantly, avoidance of splenectomy preserves optimal immunologic function against re-infection for a patient residing in an endemic area.

## 1) List of abbreviations

CT: computed tomography; ID: Infectious Disease; PCR: polymerase chain reaction.

## 2) Competing interests

The authors declare that they have no competing interests.

## 3) Authors' contributions

WT conducted the literature search, completed the chart review and authored the manuscript. DC served as a consultant for the patient, provided infectious disease input to his care and to the manuscript and also edited the manuscript. TL provided initial patient care and patient information from the outside hospital, provided information about other patients treated for Babesiosis, and also served as an editor of the manuscript. SA edited the manuscript. EM was the attending physician caring for the patient, instigated the study, edited the manuscript, and oversaw the project from start until completion. All authors read and approved the final manuscript

## 4) Consent

Written informed consent was obtained from the patient for publication of this Case report and any accompanying images. A copy of the written consent is available for review by the Editor-in-Chief of this journal
